# Supplementary material for: Comprehensive Structure-Activity Relationship Analysis of Benzamide Derivatives as Histone Deacetylase 1 (HDAC1) Inhibitors
Source: Int J Mol Sci. 2025 Oct 14;26(20):9970. doi: 10.3390/ijms26209970 (PMC12564344; doi:10.3390/ijms26209970)
Supplement: Supplementary file 1 [file ijms-26-09970-s001.zip › ijms-3750515-supplementary.pdf]

Support Information

Comprehensive Structure-Activity Relationship Analysis of Benzamide Derivatives as Histone Deacetylase 1 (HDAC1) In-hibitors

Jorge Soto-Delgado [1,*], Yeray A. Rodríguez-Núñez [2,*], Cristian Guerra [2], Luis Prent-Peñaloza [1] and Mitchell Bacho [3]

[1] Departamento de Ciencias Químicas, Facultad de Ciencias Exactas Bello, Universidad Andrés Bello, Quillota 980, Viña del Mar 2531015, Valparaíso, Chile

[2] Laboratorio de Síntesis y Reactividad de Compuestos Orgánicos, Departamento de Ciencias Químicas, Facultad de Ciencias Exactas, Universidad Andrés Bello, Republica 275, Santiago 8370146, Chile

[3] Departamento de Ciencias Biológicas y Químicas, Facultad de Medicina y Ciencia, Universidad San Sebastián, Campus Los Leones, Lota 2465, Providencia 7510085, Santiago, Chile

* Correspondence: jorge.soto@unab.cl (J.S.-D.); yeray.rodriguez@unab.cl (Y.A.R.-N.)

Table S1: Structures of benzamide analogs as HDAC1 ligands. ………………………… 2

Figure S1: RMSD evolution for (A) compound 36 (B) compound 37 (C) compound 49 (D) CPD-60 (E) CI-994 and (F) MS-275……………………………………………………… 4

Figure S2: TTClust results applied to the frames of 100 ns MD. (A) Hierarchical clustering dendrogram (B) Histogram of number of frames in each cluster and (C) distance matrix plot for compound 36……………………………………………………………………. 5

Figure S3: TTClust results applied to the frames of 100 ns MD. (A) Hierarchical clustering dendrogram (B) Histogram of number of frames in each cluster and (C) distance matrix plot for compound 37…………………………………………………………………….. 6

Figure S4: TTClust results applied to the frames of 100 ns MD. (A) Hierarchical clustering dendrogram (B) Histogram of number of frames in each cluster and (C) distance matrix plot for compound 49. …………………………………………………………………… 7

Figure S5: TTClust results applied to the frames of 100 ns MD. (A) Hierarchical clustering dendrogram (B) Histogram of number of frames in each cluster and (C) distance matrix plot for compound CPD-60……………………………………………………………. 8

Figure S6: TTClust results applied to the frames of 100 ns MD. (A) Hierarchical clustering dendrogram (B) Histogram of number of frames in each cluster and (C) distance matrix plot for compound CI-994…………………………………………………………... 9

Figure S7: TTClust results applied to the frames of 100 ns MD. (A) Hierarchical clustering dendrogram (B) Histogram of number of frames in each cluster and (C) distance matrix plot for compound MS-275…………………………………………………………. 10

Figure S8: (A) Sequence analysis of binding site for benzamide inhibitors 36 (B) superposition of HDAC1 and HCAD2 with CPD-60 benzamide inhibitor……………... 11

Table S1: Structures of benzamide analogs as HDAC1 ligands.

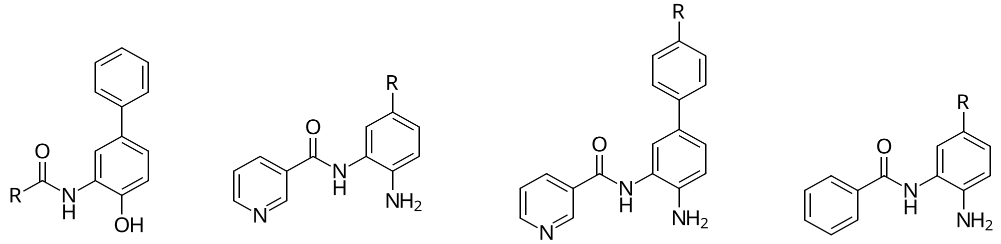

A B C D

Compd.	R	Family	IC_{50}(μM)	pIC_{50}[a]	LB1[b]	LB2[c]	RB[d]
1	Phenyl	A	0.058	7.237	6.988	6.995	6.992
2	2-Pyridyl	A	0.31	6.509	6.197	6.119	6.191
3	3-Pyridyl	A	0.068	7.168	6.910	6.860	6.914
4	4-Pyridyl	A	0.075	7.125	7.059	7.053	7.062
5	2-Thiophenyl	A	0.13	6.886	6.955	6.954	6.953
6	2-Benzothiophenyl	A	0.13	6.886	7.063	7.087	7.062
7	2-Benzothiazolyl	A	0.35	6.456	6.265	6.227	6.257
8	2-Furanyl	A	0.13	6.886	6.758	6.742	6.749
9	5-Isoxazolyl	A	0.10	7.000	6.941	6.939	6.942
10	2-Aminophenyl	A	1.8	5.744	6.030	6.002	6.030
11	3-Aminophenyl	A	0.44	6.357	6.669	6.693	6.675
12	4-Aminophenyl	A	0.040	7.398	7.024	7.033	7.026
13	Acetyl	A	4.8	5.319	5.670	5.641	5.666
14	Hydrocinnamyl	A	0.61	6.215	6.112	6.100	6.106
15	Cinnamyl	A	1.2	5.921	5.927	5.928	5.925
16	H	B	2.6	5.585	5.790	5.724	5.786
17	Phenyl	B	0.048	7.319	6.720	6.724	6.724
18	2-Thiophenyl	B	0.065	7.187	7.182	7.192	7.188
19	3-Thiophenyl	B	0.052	7.284	7.143	7.199	7.141
20	3-Furanyl	B	0.16	6.796	7.045	7.076	7.034
21	1-Imidazolyl	B	1.0	6.000	6.073	6.138	6.094
22	4-Pyridyl	B	0.65	6.187	6.642	6.642	6.650
23	1-Pyrrolidinyl	B	0.67	6.174	6.558	6.317	6.547
24	Cyclopentyl	B	4.2	5.377	6.035	5.746	6.473
25	F	C	0.14	6.854	6.656	6.582	6.655
26	NH_2	C	0.33	6.482	6.182	6.209	6.188
27	NMe_2	C	5.3	5.276	5.120	5.124	5.122
28	CH_2CN	C	0.25	6.602	6.299	6.257	6.308
29	CH_2NH_2	C	2.1	5.678	5.755	5.750	5.760
30	CO_2CH_2Ph	C	1.3	5.886	5.247	5.106	5.230
31	$CH_2CH_2CO_2H$	C	13.0	4.886	4.807	4.754	4.801
32	$CH_2CH_2CO_2Me$	C	1.5	5.824	5.656	5.663	5.656
33	H	D	2.4	5.620	5.870	5.864	5.862
34	Phenyl	D	0.060	7.222	6.826	6.880	6.825
35	2-Thiophenyl	D	0.048	7.319	6.448	6.377	6.436

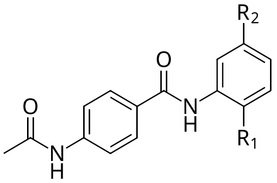

E

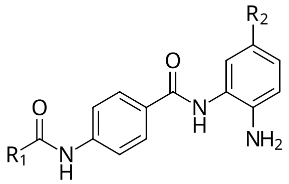

F

Compd.	R_1	R_2	Family	IC_{50}(μM)	pIC_{50}[a]	LB[b]		RB[c]
36	OH	Ph	E	0.018	7.745	7.670	7.630	7.662
37	NH_2	Ph	E	0.028	7.553	7.750	7.740	7.734
38	NH_2	3-Thiophene	E	0.007	8.155	7.803	7.820	7.789
CPD-60	NH_2	2-Thiophene	E	0.007	8.155	7.644	7.704	7.621
CI-994	NH_2	H	E	0.57	6.244	6.373	6.304	6.351
39	3-Pyridyl-CH_2O	Ph	F	0.010	8.000	8.235	8.263	8.232
40	3-Pyridyl-$(CH2)_2$	Ph	F	0.011	7.958	8.157	8.179	8.163
41	2-Pyridyl-CH_2O	Ph	F	0.008	8.096	7.647	7.560	7.652
42	BenzylO	Ph	F	0.017	7.769	7.841	7.832	7.838
43	PhenylO	Ph	F	0.016	7.795	7.621	7.587	7.625
44	EthylO	Ph	F	0.013	7.886	7.934	7.973	7.935
45	MethylO	Ph	F	0.010	8.000	7.818	7.836	7.817
46	n-Propyl	Ph	F	0.010	8.000	8.013	8.059	8.018
47	Ethyl	Ph	F	0.010	8.000	7.909	7.935	7.912
48	Cyclohexyl	Ph	F	0.020	7.698	8.020	8.068	8.025
49	3-Pyridyl-CH_2O	2-Thiophenyl	F	0.006	8.221	8.241	8.257	8.245
50	EthylO	2-Thiophenyl	F	0.007	8.155	8.355	8.392	8.361
51	MethylO	2-Thiophenyl	F	0.006	8.221	8.605	8.594	8.608
52	n-Propyl	2-Thiophenyl	F	0.009	8.045	7.741	7.688	7.734
53	Ethyl	2-Thiophenyl	F	0.009	8.045	8.342	8.367	8.349
54	Cyclohexyl	2-Thiophenyl	F	0.013	7.886	7.670	7.617	7.662
MS-275	3-Pyridyl-CH_2O	H	F	0.19	7.721	7.271	7.240	7.261

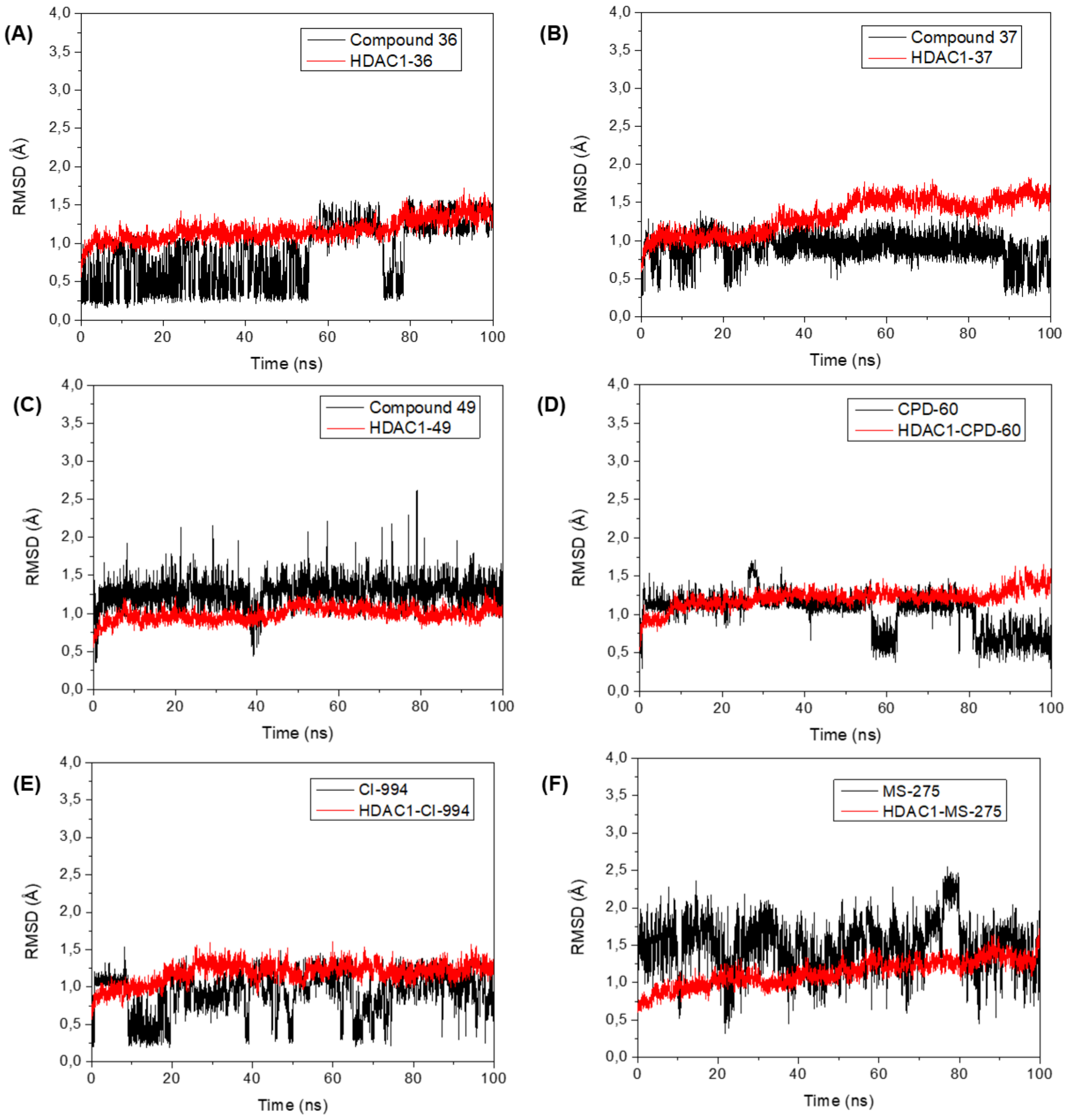

Figure S1: RMSD evolution for (A) compound 36 (B) compound 37 (C) compound 49 (D) CPD-60 (E) CI-994 and (F) MS-275.

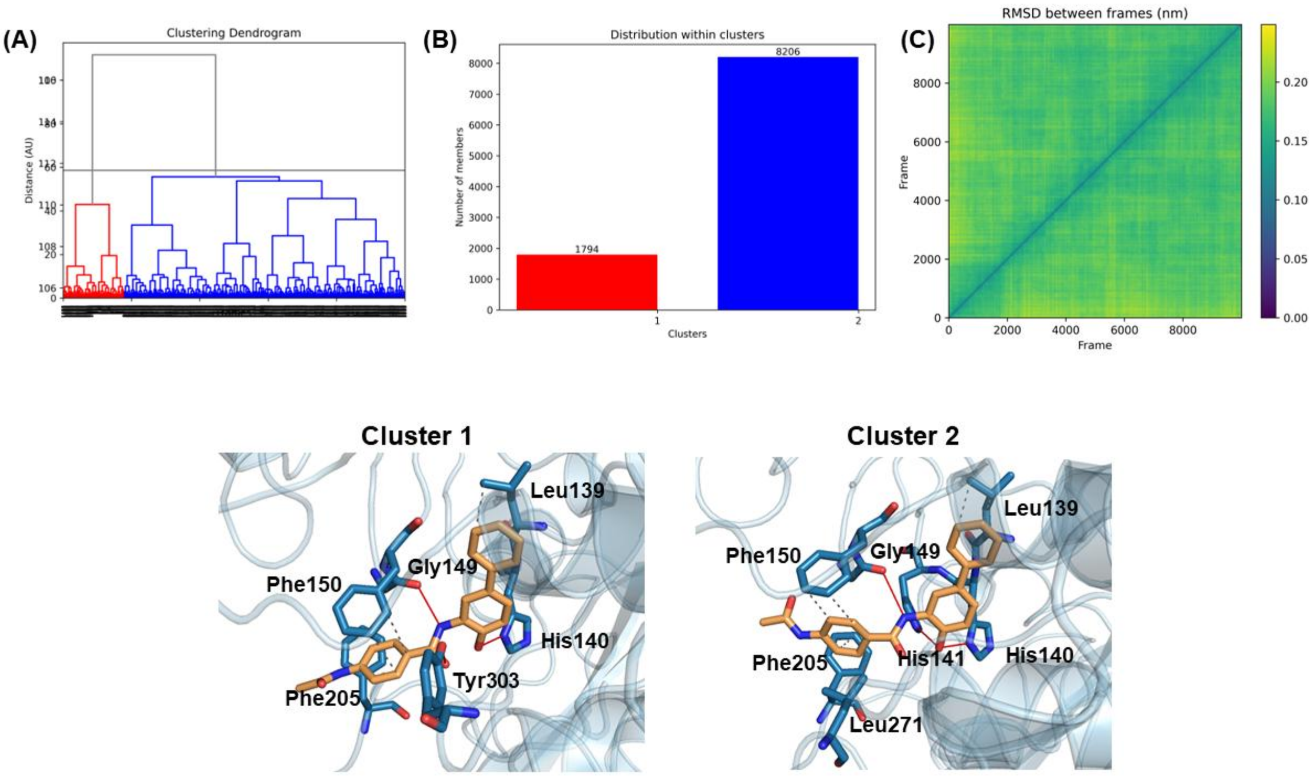

Figure S2: TTClust results applied to the frames of 100 ns MD. (A) Hierarchical clustering dendrogram (B) Histogram of number of frames in each cluster and (C) distance matrix plot for compound 36.

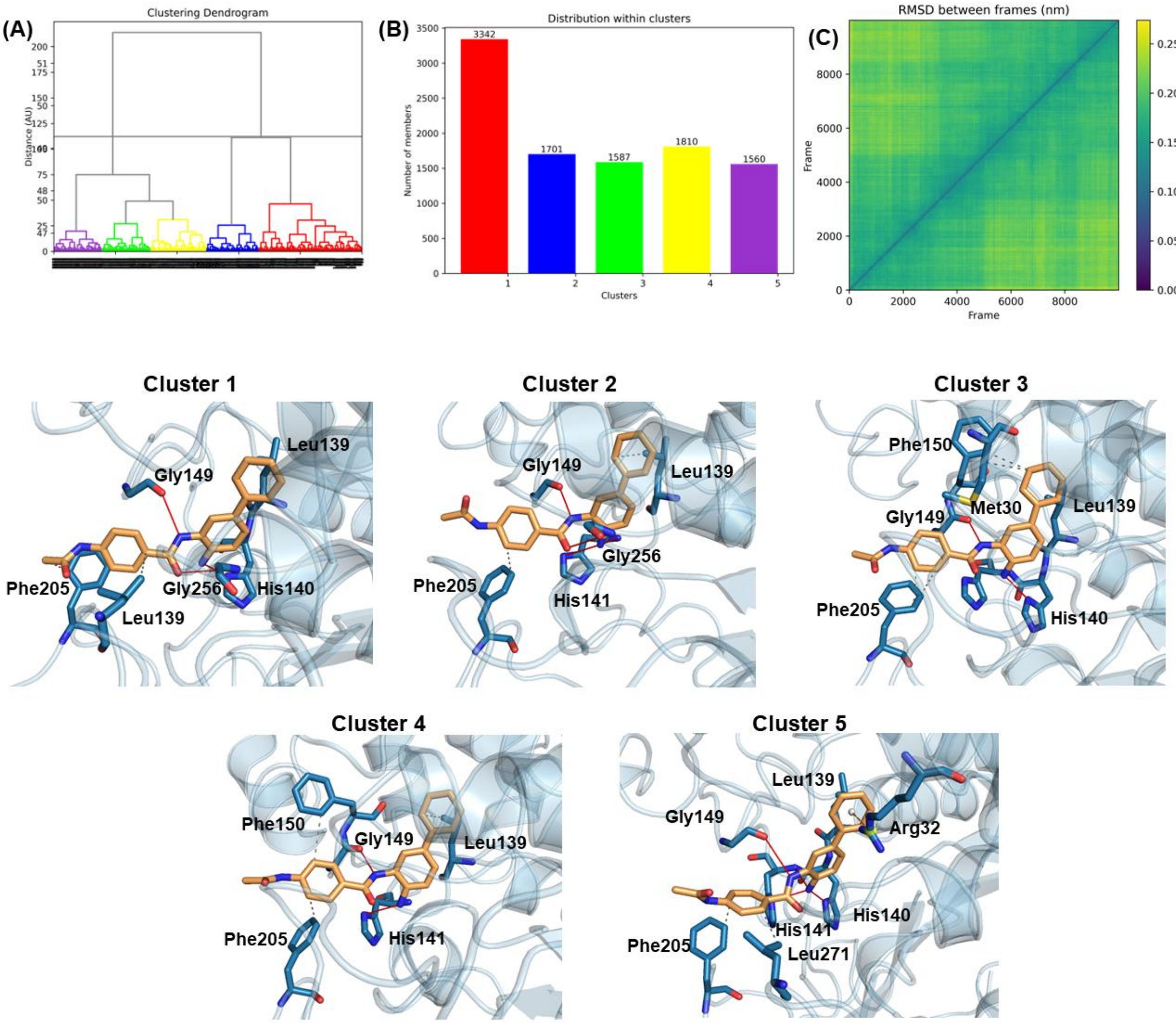

Figure S3: TTClust results applied to the frames of 100 ns MD. (A) Hierarchical clustering dendrogram (B) Histogram of number of frames in each cluster and (C) distance matrix plot for compound 37.

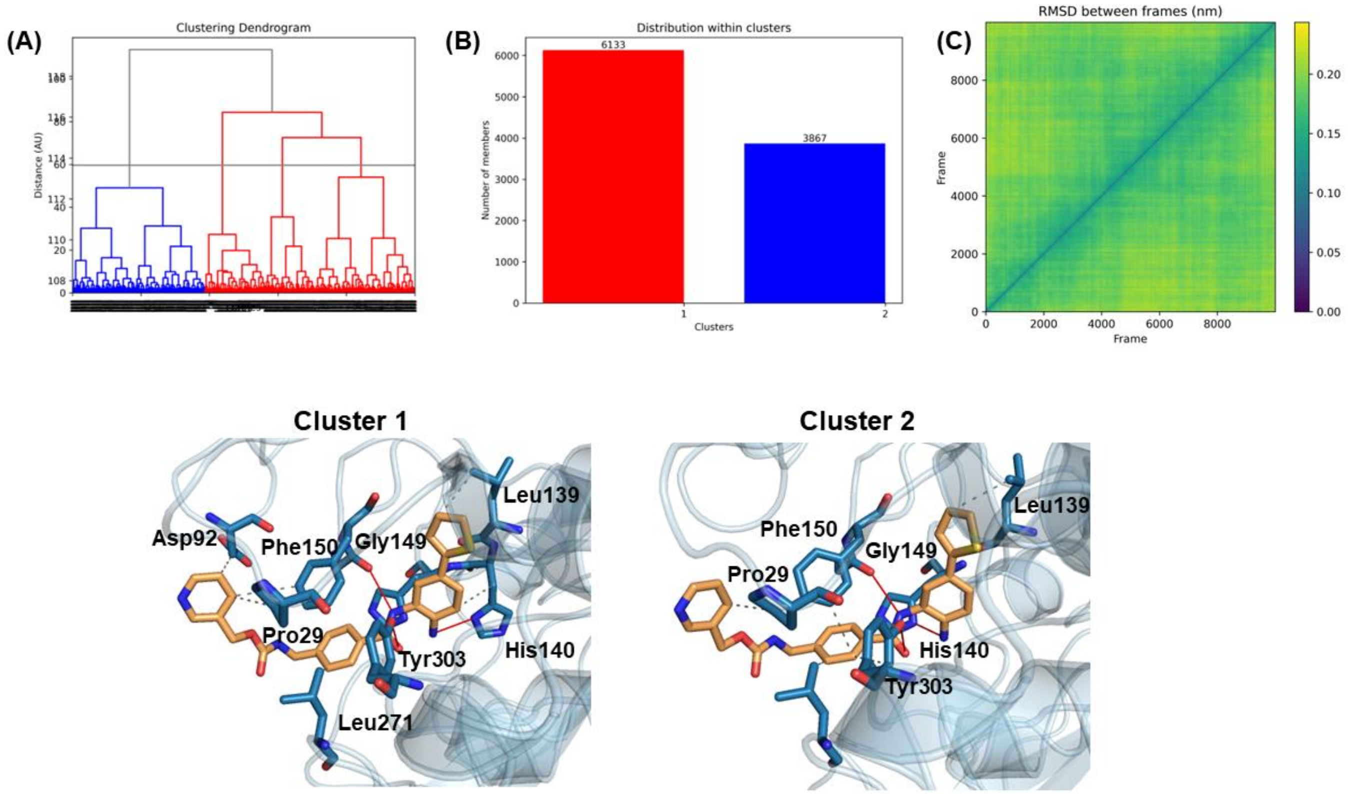

Figure S4: TTClust results applied to the frames of 100 ns MD. (A) Hierarchical clustering dendrogram (B) Histogram of number of frames in each cluster and (C) distance matrix plot for compound 49.

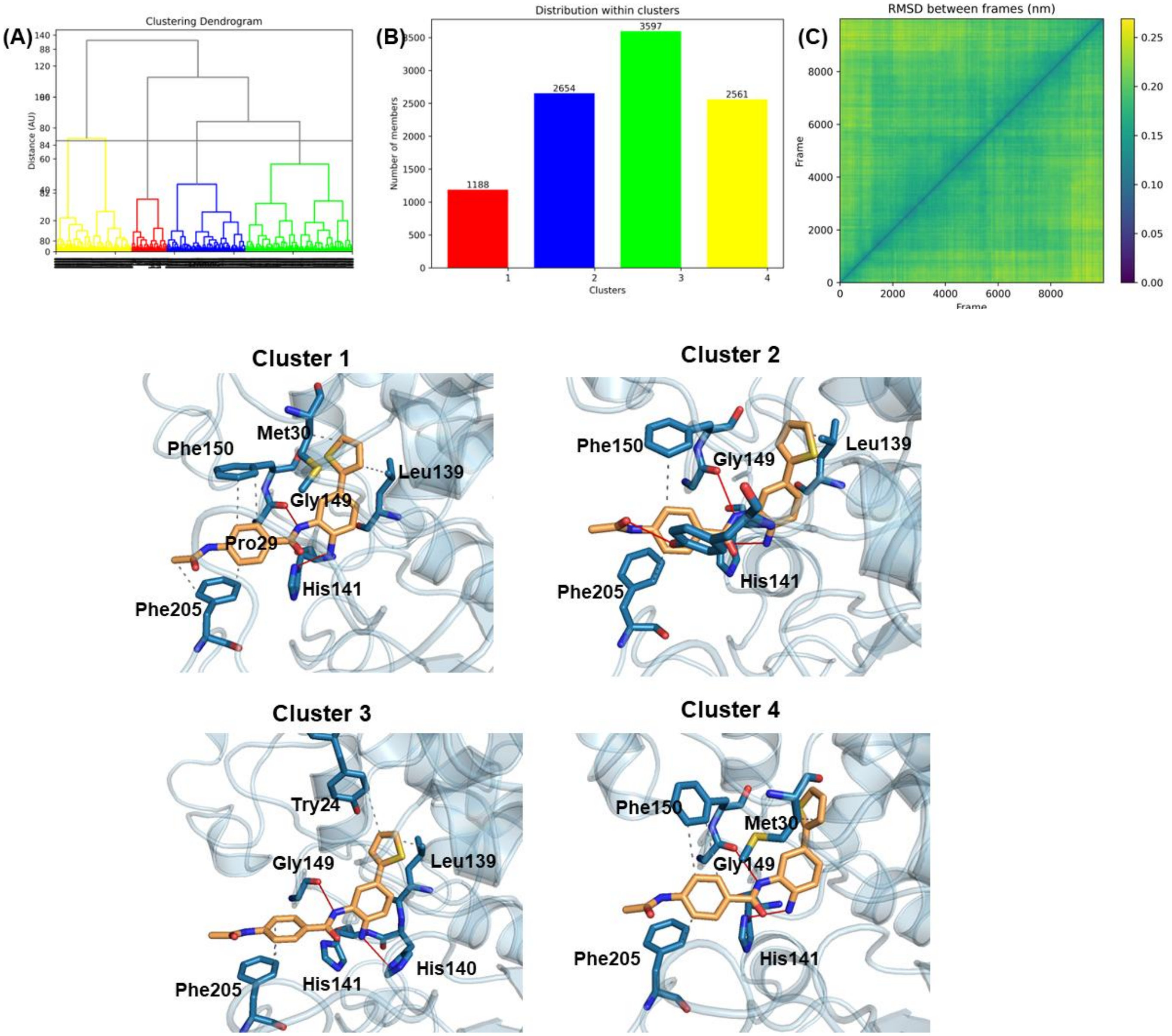

Figure S5: TTClust results applied to the frames of 100 ns MD. (A) Hierarchical clustering dendrogram (B) Histogram of number of frames in each cluster and (C) distance matrix plot for compound CPD-60.

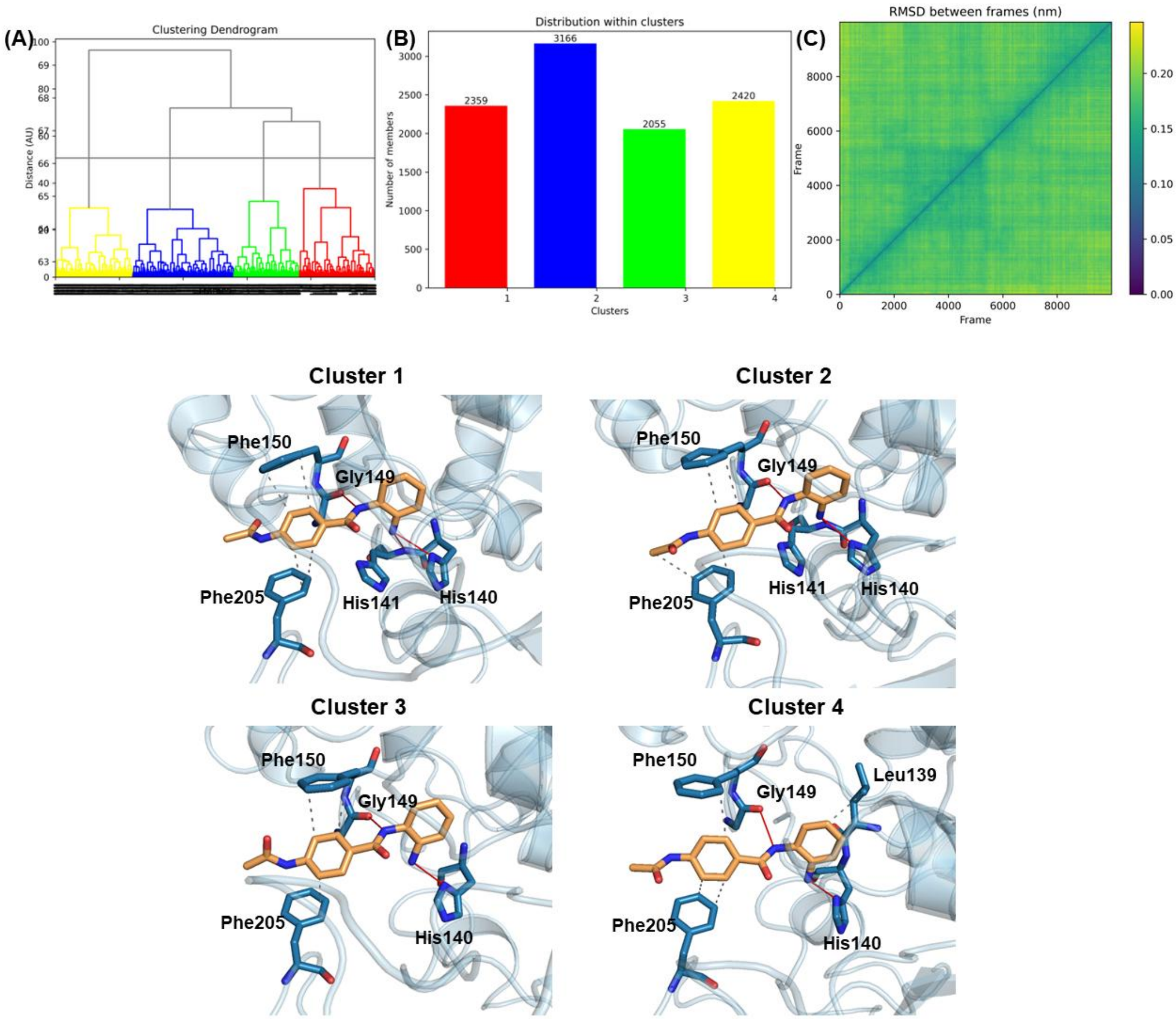

Figure S6: TTClust results applied to the frames of 100 ns MD. (A) Hierarchical clustering dendrogram (B) Histogram of number of frames in each cluster and (C) distance matrix plot for compound CI-994.

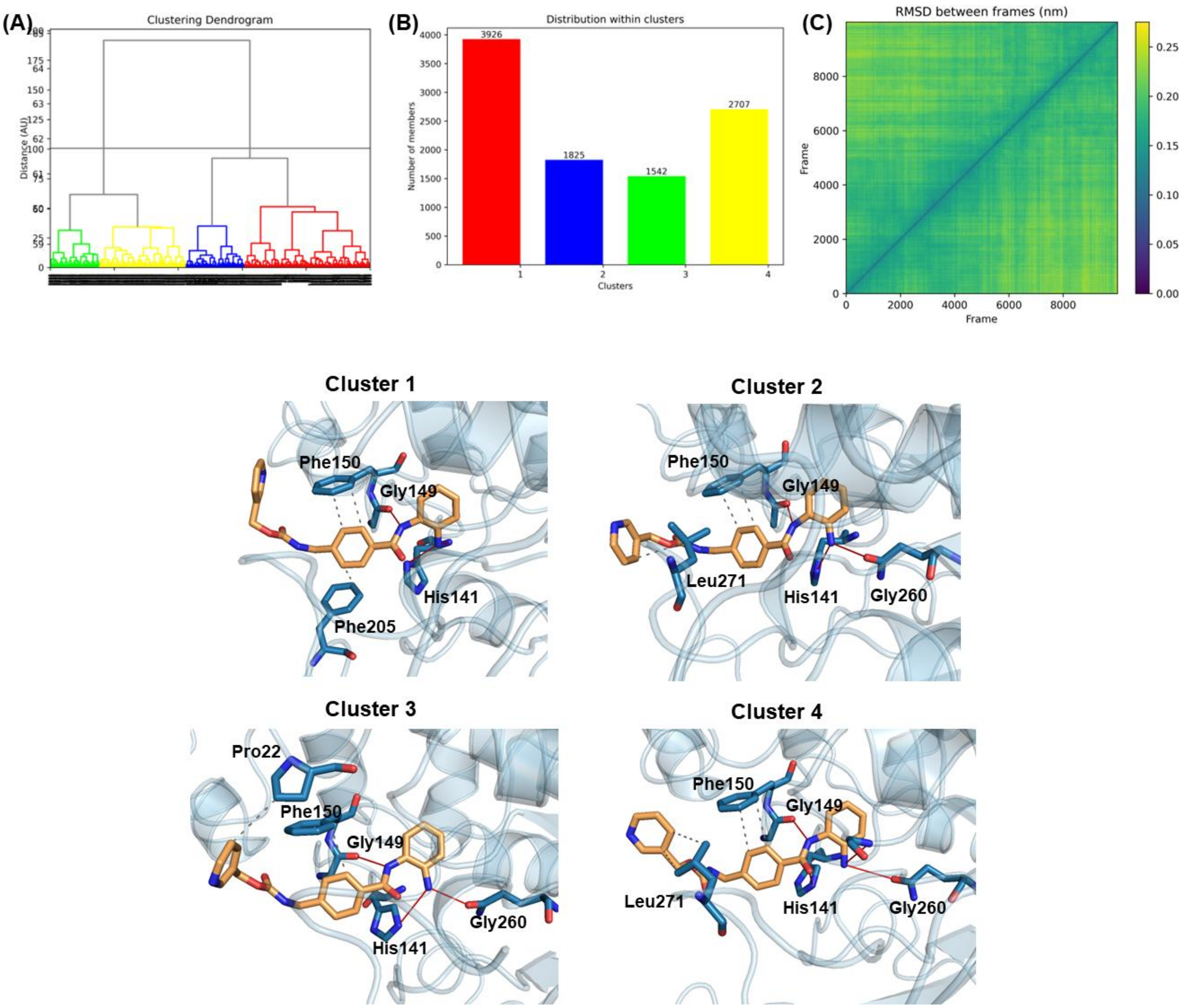

Figure S7: TTClust results applied to the frames of 100 ns MD. (A) Hierarchical clustering dendrogram (B) Histogram of number of frames in each cluster and (C) distance matrix plot for compound MS-275.

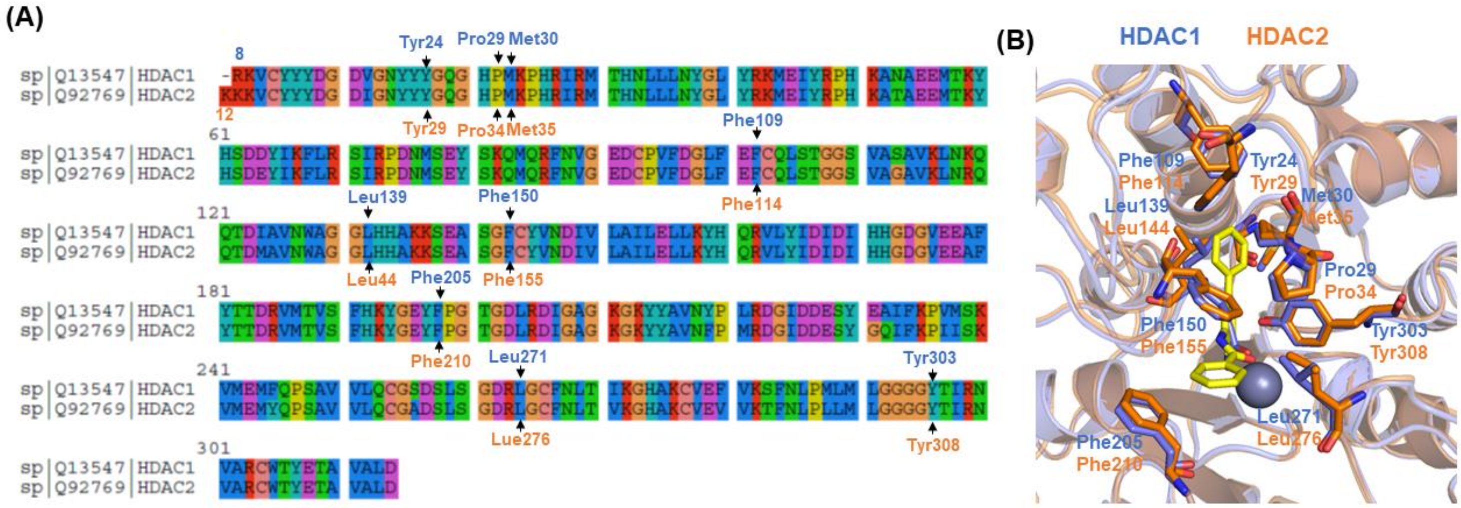

Figure S8: (A) Sequence analysis of binding site for benzamide inhibitors 36 (B) superposition of HDAC1 and HCAD2 with CPD-60 benzamide inhibitor
